# Exposing an “Intangible” Cognitive Skill among Collegiate Football Players: Enhanced Interference Control

**DOI:** 10.3389/fpsyg.2018.00049

**Published:** 2018-02-09

**Authors:** Scott A. Wylie, Theodore R. Bashore, Nelleke C. Van Wouwe, Emily J. Mason, Kevin D. John, Joseph S. Neimat, Brandon A. Ally

**Affiliations:** ^1^Department of Neurosurgery, University of Louisville, Louisville, KY, United States; ^2^Department of Psychology, University of Northern Colorado, Greeley, CO, United States; ^3^Department of Neurology, Vanderbilt University Medical Center, Nashville, TN, United States

**Keywords:** interference control, flanker task, football, inhibition, attention

## Abstract

American football is played in a chaotic visual environment filled with relevant and distracting information. We investigated the hypothesis that collegiate football players show exceptional skill at shielding their response execution from the interfering effects of distraction (*interference control*). The performances of 280 football players from National Collegiate Athletic Association Division I football programs were compared to age-matched controls in a variant of the Eriksen flanker task (Eriksen and Eriksen, 1974). This task quantifies the magnitude of interference produced by visual distraction on split-second response execution. Overall, football athletes and age controls showed similar mean reaction times (RTs) and accuracy rates. However, football athletes were more proficient at shielding their response execution speed from the interfering effects of distraction (i.e., smaller flanker effect costs on RT). Offensive and defensive players showed smaller interference costs compared to controls, but defensive players showed the smallest costs. All defensive positions and one offensive position showed statistically smaller interference effects when compared directly to age controls. These data reveal a clear cognitive advantage among football athletes at executing motor responses in the face of distraction, the existence and magnitude of which vary by position. Individual differences in cognitive control may have important implications for both player selection and development to improve *interference control* capabilities during play.

## Introduction

Football is played in a dynamic motoric and visual environment. The motor (i.e., physical) skills required to play the game at a high level are readily evident, even to the most inexperienced observer. In contrast, the cognitive skills that are foundational to the successful expression of these physical skills are not so evident, even to the most experienced observer. Indeed, among football coaches, analysts, and enthusiasts, these cognitive skills are commonly viewed as mysterious, elusive, and probably immeasurable. Hence, in the common vernacular of football, they are typically referred to as the intangible or instinctual elements of a player’s skill set. These are precisely the kinds of cognitive skills that can be exposed, however, using the experimental tools available to cognitive scientists. During performance, football players are bombarded with both relevant and distracting stimulus information that has the potential to compromise their response execution. These divergent sources of information place considerable demands on executive cognitive control systems that are crucial to focusing and executing split-second reactions while controlling interference from distracting information and the conflicting responses it elicits (i.e., *interference control*). Here, we report an investigation of the hypothesis that high-level college football players are more proficient at *interference control* than are young adults from the general university population who do not play collegiate athletics.

A rich history in the cognitive sciences demonstrates that the mere presence of distracting stimulus information in the visual field can interfere with motor reactions, particularly if the stimulus information is strongly associated with conflicting responses. Among the most elegant experimental procedures for quantifying the degree to which distracting stimulus information in the visual environment interferes with response execution is the Eriksen flanker task (Eriksen and Eriksen, 1974). In this task, participants make a series of speeded choice reactions to a target stimulus that is flanked on each side by distracting stimuli (i.e., flankers). For example, participants can be instructed to issue a left- or a right-hand response to, respectively, a left- or right-pointing target arrow that is flanked by distracting arrows that point either in the same (i.e., congruent) or in the opposite (i.e., incongruent) direction as the target. Despite their irrelevance to the target-decision response, flanker arrows pointing in the opposite direction as the target slow RT and increase response errors compared to flanker arrows pointing in the same direction as the target. This phenomenon is referred to as the *flanker effect*. A vast literature indicates that the flankers are processed automatically and in parallel with the controlled processing of the target, and converges on support for a dual-process conceptualization of these processes (Ridderinkhof et al., 1995; Ridderinkhof, 2002; Mattler, 2005; van den Wildenberg et al., 2010). Within the context of this conceptualization, the faster, automatic processing of the flankers produces an initial activation of the response signaled by the flankers that either facilitates the reaction signaled by the target (i.e., the same response) or conflicts with the reaction signaled by the target (i.e., the opposite response). The flanker-driven response is expressed neurophysiologically by early activation of the motor cortex contralateral to the response side signaled by the flankers as well as electromyographically by patterns of subthreshold muscle activations in the response forearm and hand signaled by the flankers (Gratton et al., 1988; Burle et al., 2014; Klein et al., 2014). In the case of conflict, activation of the incorrect response interferes with activation of the correct response and must be suppressed by cognitive control mechanisms so that the correct response can be executed and premature response errors can be prevented (van den Wildenberg et al., 2010). Suppression grows in strength gradually over time and is effectuated late in processing when the response selection decision is being made. Thus, the magnitude of the flanker effect exposes the proficiency of an individual’s cognitive control system at resolving interference produced by response conflict.

The considerable individual and group differences revealed in studies of *interference control* in visual environments supports the conclusion that there exists a wide range of skill in controlling motor system interference produced by distracting, response-activating visual information (Hazeltine et al., 2000; Botvinick et al., 2001). Small performance costs when executing responses in the face of incongruent flankers are attributed to an extremely proficient *interference control* system capable of maintaining highly-focused attention and response execution speed while effectively suppressing interfering response tendencies triggered in the motor system. In contrast, large costs on performance speed and accuracy in the face of incongruent flankers are suggestive of pronounced difficulty filtering visual distraction and suppressing the resulting interference produced by the activation of incorrect response tendencies. Thus, performance in the flanker task provides direct measures, response speed and accuracy, of an individual’s proficiency at *interference control* in a visually distracting environment. Given the sensitivity of these measures, the flanker task has been used extensively to quantify striking reductions in *interference control* caused by neurological and psychiatric disorders, such as Parkinson’s disease (e.g., Wylie et al., 2009a,b; van Wouwe et al., 2014) and attention deficit hyperactivity disorder (e.g., Ridderinkhof et al., 2005; Mullane et al., 2009), that are associated with alterations to frontal-basal ganglia circuitries.

Unlike the large literature that exists of diminished *interference control* in patient populations, there is essentially no research literature devoted to studying this type of control in populations, like highly-skilled athletes, who could be expected to have superior control. However, consistent with the call of Voss et al. (2010), there is an emerging literature in which RT tasks of varying levels of complexity have been used to characterize potentially exceptional neurocognitive capacity in highly-skilled athletes whose performance expertise is expressed in dynamic visual environments (e.g., [*baseball*: Muraskin et al., 2015; Yamashiro et al., 2015] [*football*: Solomon et al., 2013] [*martial arts*: Mori et al., 2002; Sanchez-Lopez et al., 2014; Chang et al., 2017] [*rugby*: Mori and Shimada, 2013] [*soccer*: Vestberg et al., 2012; Verburgh et al., 2014] [*team handball*: Memmert et al., 2009] [*tennis*: Wang et al., 2013] [*volleyball*: Alves et al., 2013]). Within this body of research, we are aware of only one study in which differences in *interference control* between athletes and controls have been assessed in a flanker task (Wang et al., 2017; see Kokubu et al., 2006, for use of an alternative task to assess *interference control* in volleyball players). Using an arrow variant of the flanker task, Wang et al. (2017) found that professional badminton players were better able to inhibit the influence of incongruent flankers than were highly-trained competitive athletes who did not compete in visually-dynamic sports (track and field, dragon boat racing).

Given the demands made on the *interference control* capacity of a football player during a game, the well-characterized sensitivity of the flanker task to demands of this type, and the paucity of research assessing this capacity in highly-skilled athletes, we undertook the current investigation. It addressed three key aims. First, we tested the hypothesis that football players playing at the top collegiate level show more proficient *interference control* during response execution than do their age peers in the general student population who do not participate in extramural collegiate athletics. Because the extant literature comprises studies that report response speeds among highly-skilled athletes who perform in visually-dynamic environments that are either faster than (e.g., Mori et al., 2002; Kokubu et al., 2006; Mori and Shimada, 2013; Wang et al., 2013; Verburgh et al., 2014; Muraskin et al., 2015) or comparable to (e.g., Memmert et al., 2009; Sanchez-Lopez et al., 2014; Chang et al., 2017) those of various types of controls (e.g., track and field athletes, novice athletes, non-athlete controls), we predicted, conservatively, that the overall mean RTs of football players and general student controls would not differ but, like badminton players and given the visual processing demands of their sport, football players would have superior skill at filtering the interfering effects of visual distraction on response execution (i.e., smaller performance costs produced by incongruent flankers). Second, we compared offensive and defensive players in their capacity to control interference effects. This comparison was informed by our perspective that even though *interference control* is undoubtedly a crucial cognitive skill for all players on the field, demands on it may be greater for defensive players who are reacting to the efforts of offensive opponents to create visual distraction through misdirection and conflict (e.g., ball fakes, crossing routes, misdirection counter plays). In other words, effectiveness as a defensive player may require a higher level of *interference control* because of the unpredictable and disruptive visual chaos created by opponents on virtually every play. Third, we conducted an exploratory analysis of differences in *interference control* between different offensive and defensive positions. We reasoned that this would be a critical first step in determining the extent to which *interference control* is an essential component of the cognitive skill set needed to meet the unique demands of specific football positions.

## Materials and Methods

### Participants

Data were collected from 280 male collegiate football athletes (mean age 19.9 ± 1.6) and 35 male controls from the general student population (mean age 20.3 ± 2.6)^1^. Football athletes were all current roster players recruited from five National Collegiate Athletic Association (NCAA) Division I football programs. Controls were recruited from the general university population at the University of Northern Colorado and interviewed to confirm no history of participation in collegiate sports. None of the football athletes were in an active concussion protocol at the time of testing or had experienced a blow to the head that kept them from physical activity within the 3 months prior to testing. Controls had no history of head injury. All participants had normal or corrected-to-normal vision, as indicated by self-report. This study and consenting procedures were reviewed and approved by the Institutional Review Boards at the University of Louisville and University of Northern Colorado.

### Flanker Task and Procedures

The *flanker task* was administered on a MacMini with a 17-inch Dell monitor placed approximately 1 m in front of the participant. The task was programmed and administered using PsychToolbox and Matlab software tools (MathWorks, 2014), which interfaced with an RB series response button box to register responses with 2–3 ms reaction time (RT) resolution (Cedrus, Incorporated^2^). The beginning of the task was signaled by the appearance of a small, centrally-located white fixation square (0.5 cm sides) against a dark gray-colored screen for 250 ms. Next, the fixation square was extinguished and replaced by a horizontal array of five arrows measuring 1.5 cm high by 7 cm wide. The center arrow of the array appeared in the same location as the fixation square and was flanked on each side by two arrows (e.g., 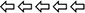). The arrow array remained on the screen for 250 ms and then disappeared. Participants had 1000 ms to respond to the direction of the center arrow before the trial ended. A variable intertrial interval of 800–1000 ms elapsed before the next trial was initiated by the appearance of another arrow array. During this interval, the fixation square re-appeared on the screen (i.e., at the offset of the arrow array). The end of the task was indicated by the offset of the fixation square and the appearance of printed instructions, centered on the computer screen, that the task was completed.

Participants were instructed to respond in the direction indicated by the center arrow in the array (i.e., the target arrow). Specifically, they were instructed to press the button under their left index finger to a center arrow pointing to the left and under their right index finger to a center arrow pointing to the right. The response device was positioned in front of the participant so that the left and right index fingers were placed, respectively, on the far left and far right response buttons of a horizontal seven-button panel. Left- and right-pointing center arrows appeared pseudo-randomly; that is, with the constraint that they appeared with equiprobability across the task. Participants were encouraged to focus visual attention on the fixation square and to respond as quickly and as accurately as possible when the target arrow appeared (i.e., balance speed with accuracy). To elicit the flanker effect, we varied the directions the center and flanking arrows pointed on a trial-by-trial basis. This factor, *Flanker Type*, had two levels, *Congruent (Cg)* and *Incongruent (Ig)*. On *Cg* trials, the flankers pointed in the same direction as the center target (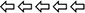), thus signaling the same response hand. On *Ig* trials, the flankers pointed in the opposite direction of the center target (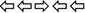), thus signaling a conflicting response to the target. *Cg* and *Ig* flanker types occurred pseudo-randomly throughout the task, as was the case for arrow direction. In total, participants completed 30 practice trials followed by 100 experimental trials, equally divided between the two flanker types.

### Data Analyses

Mean RTs were calculated for correct response trials across *Cg* and *Ig* trials. We also calculated mean accuracy rates separately for the two trial types. However, because accuracy rates are not normally distributed in choice reaction tasks, we analyzed means of square root-transformed accuracy rates (McDonald, 2014). Interference (i.e., flanker) effects were derived by subtracting mean RT and accuracy rates for *Cg* trials from *Ig* trials. Smaller flanker effects on RT and accuracy rates indicate higher proficiency at interference control (i.e., reduced interference from distraction on response execution). These measures were first analyzed separately using repeated-measures analysis of variance (ANOVA) to determine the main and interactive effects of *Flanker Type* (*Cg*, *Ig*) and *Group* (football athlete, general student control). In these analyses, degrees of freedom were 1 and 313. We then re-analyzed the dependent measures within the football athletes to assess the effect of *General Position* (Offense, Defense) followed by more specific analyses to address potential performance differences in *Specific Positions* within each group of offensive (Quarterback [QB, *n* = 26], Running Back [RB, *n* = 30], Wide Receiver/Tight End [WR/TE, *n* = 50], Offensive Lineman [OL, *n* = 53]) and defensive (Defensive Lineman [DL, *n* = 40], Linebacker [LB, *n* = 30], Defensive Back [DB, *n* = 42]) player groups. Special teams players, such as Punters (*n* = 3) and Kickers (*n* = 6), were excluded from the analysis of offensive versus defensive and specific position effects. In the last two sets of analyses, the degrees of freedom were, respectively, 1 and 269 and 7 and 298, unless otherwise specified.

## Results

### Comparison of Collegiate Football Players and Controls

**FIGURE 1:**
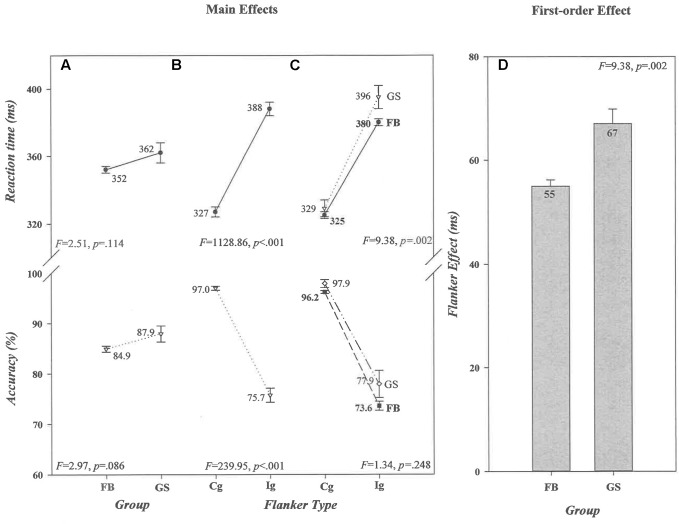
Absence of main effects of *Group* and presence of main effects of *Flanker Type* for reaction time (RT) (in ms) and accuracy (in percent correct) are shown, respectively, in **(A,B)**. The values associated with each data point are shown by each point, and the associated *F* ratios and *p*-values for RT and accuracy for each factor in the ANOVA are shown in each panel. The size differences in the Flanker Effect (in ms) that produced the significant *Group* × *Flanker Type* interaction are illustrated in **(C,D)**. In **(D)**, the absolute size (in ms) of the effect for each group is shown in the bar for that group. Error bars represent the standard error of the mean. FB, football athletes; GS, general student control; Cg, congruent flanker type; Ig, incongruent flanker type.

As can be seen in panel A of **Figure 1**, overall mean RTs and accuracy rates did not differ between football athletes (352 ms, 84.9%) and controls (362 ms, 87.9%) (*Group* [RT, *F* = 2.51, *p* = 0.114, $\eta_{p}^{2}$ = 0.008] [Acc, *F* = 2.97, *p* = 0.086, $\eta_{p}^{2}$ = 0.009]). It can also be seen in panel B that a very robust flanker effect was produced on both dependent measures (*Flanker Type* [RT, *F* = 1128.86, *p* < 0.001, $\eta_{p}^{2}$ = 0.783] [Acc, *F* = 239.95, *p* < 0.001, $\eta_{p}^{2}$ = 0.434]). Responses were 61 ms slower and 21% less accurate on *Ig* than on *Cg* trials. However, the size of the flanker effect, illustrated in panels C and D, did differ between the two groups for RT, but not for accuracy (*Group* × *Flanker Type* [RT, *F* = 9.38, *p* = 0.002, $\eta_{p}^{2}$ = 0.029] [Acc, *F* = 1.34, *p* = 0.248, $\eta_{p}^{2}$ = 0.004]). The RT effect was significantly smaller among football athletes (55 ms) than controls (67 ms; *t*(313) = -3.04, *p* = 0.003, $\eta_{p}^{2}$ = 0.029). Thus, visual distractors signaling conflicting motor responses had significantly less impact on the response execution speed of football athletes than of controls, suggesting more proficient *interference control* among football athletes.

### Comparison of Offensive and Defensive Football Player Groups

**FIGURE 2:**
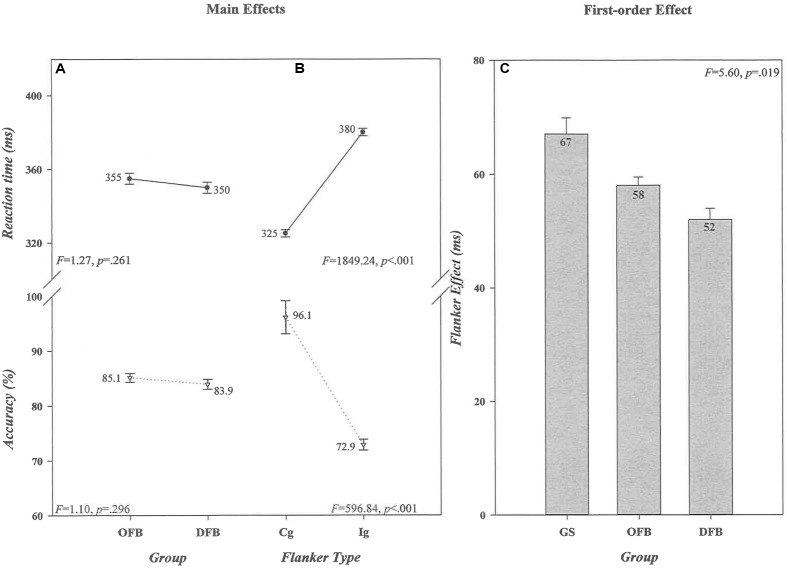
Absence of main effects of *Group* and presence of main effects of *Flanker Type* for RT (in ms) and accuracy (in percent correct) for the offensive and defensive football players comparison are shown, respectively, in **(A,B)**. The values associated with each data point are shown by each point, and the associated *F* ratios and *p*-values for RT and accuracy for each factor in the ANOVA are shown in each panel. The size differences in the Flanker Effect (in ms) that produced the significant *Group* × *Flanker Type* interaction when offensive and defensive players were compared against general student controls are illustrated in **(C)**. The absolute size of the effect (in ms) for each group is shown in the bar for that group. Error bars represent the standard error of the mean. OFB, offensive football players; DFB, offensive football players; GS, general student control; Cg, congruent flanker type; Ig, incongruent flanker type.

The overall mean response speeds and accuracies of offensive (*n* = 159 [355 ms, 85.1%]) and defensive (*n* = 112 [350 ms, 83.9%]) players, illustrated in **Figure 2A**, were comparable (*Group* [RT, *F* = 1.27, *p* = 0.261, $\eta_{p}^{2}$ = 0.005] [Acc, *F* = 1.10, *p* = 0.296, $\eta_{p}^{2}$ = 0.004]). As was the case in the comparison between football athletes and controls, a very robust flanker effect, depicted in **Figure 2B**, was evident in this comparison (*Flanker Type* [RT, *F* = 1849.24, *p* < 0.001, $\eta_{p}^{2}$ = 0.873] [Acc, *F* = 596.84, *p* < 0.001, $\eta_{p}^{2}$ = 0.689]). Responses were 55 ms slower and 23% less accurate on *Ig* than on *Cg* trials. Of greatest interest, however, is that the magnitude of the flanker effect differed between offensive and defensive players on RT, but not on accuracy (*Position* × *Flanker Type* [RT, *F* = 5.60, *p* = 0.019, $\eta_{p}^{2}$ = 0.020] [Acc, *F* = 0.16, *p* = 0.689, $\eta_{p}^{2}$ = 0.001]). Specifically, the magnitude of the effect on RT was significantly smaller among defensive (52 ms) than offensive (58 ms) players [*t*(269) = 2.35, *p* = 0.020, $\eta_{p}^{2}$ = 0.020]. As depicted in **Figure 2C**, a separate ANOVA comparing player groups to controls revealed that the flanker effects on RT were smaller in offensive and defensive players than in controls [*Group*, *F*(2,303) = 7.58, *p* = 0.001, $\eta_{p}^{2}$ = 0.048; Dunnett’s *post hoc* tests: Offensive vs. Controls, *p* = 0.018, Defensive vs. Controls, *p* < 0.001]. Thus, visual distractors signaling conflicting motor responses produced significantly less interference on response execution speed in both offensive and defensive football players than in controls, but defensive players were more proficient than offensive players at controlling this interference.

### Comparison of Offensive and Defensive Football Position Groups

**FIGURE 3:**
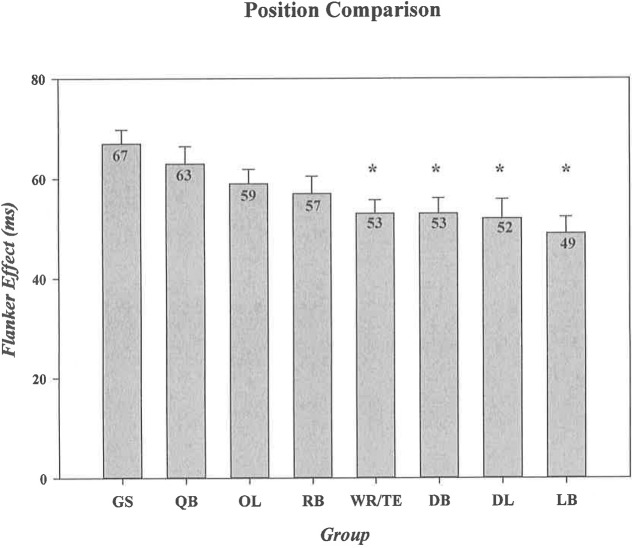
Comparative sizes of the Flanker Effect in general student controls and in football athletes differentiated by position. ^∗^ Indicates a statistically significant difference between a particular position group and the general student control group. Error bars represent the standard error of the mean. GS, general student control; QB, quarterback; OL, offensive lineman; RB, running back; WR/TE, wide receiver/tight end; DB, defensive back; DL, defensive lineman; LB, linebacker. The absolute size of the effect (in ms) for each group is shown in the bar for that group.

In an exploratory analysis, we compared specific offensive (QB, RB, WR/TE, OL) and defensive (DL, LB, DB) positions against the control group to determine if specific groups of players tasked with unique demands on the football field display advantages in *interference control*. We computed separate one-way ANOVAs for the magnitudes of flanker effects on RT and on response accuracy. Flanker effects varied across subgroups only on RT, as shown in **Figure 3** [*Group*, *F*(7,298) = 2.93, *p* = 0.006, $\eta_{p}^{2}$ = 0.064]. Dunnett’s *post hoc* comparisons to the control group revealed that one offensive position group (WR/TE *p* = 0.011) and all three defensive position groups (DL *p* = 0.007; LB *p* = 0.002; DB *p* = 0.015) had significantly smaller flanker effects on RT. Flanker effects on accuracy did not vary by subgroup, although the *F*-statistic narrowly missed the significance threshold (*Group*, *F* = 1.99, *p* = 0.056, $\eta_{p}^{2}$ = 0.045). Notably, an exploratory Dunnett’s *post hoc* comparison of football position groups to the control group did not reveal any position group differences in accuracy effects (all comparisons to the control yielded *p*s > 0.78).

## Discussion

The flanker task produced highly robust performance effects consistent with a longstanding literature (Eriksen and Eriksen, 1974; Mattler, 2005). On average, RT slowed by 61 ms and response execution errors increased by 21% when participants issued quick reactions to a target stimulus that was flanked by distractors signaling a response that conflicted with the response signaled by the target (i.e., flanker effect). Collegiate football players and age peers selected from the general student body reacted with similar overall mean RTs and accuracy rates. Critically, however, collegiate football players showed a significantly reduced flanker effect on RT compared to their age peers. Thus, response execution among football players was less susceptible to conflict from distracting stimulus information that activates conflicting response tendencies, suggesting they possess enhanced *interference control* capabilities. This enhanced control was evident in both offensive and defensive position players, although defensive players showed significantly better *interference control* than offensive players. Finally, all three defensive position groups (DL, LB, DB), but only one offensive position group (WR/TE), showed better *interference control* skills than their age peers when contrasted directly with them.

Our results indicate that when making response execution decisions collegiate football players are, as a group, more proficient than their age peers at shielding these decisions from the interfering effects of visual distractions and the conflicting response tendencies they activate. In a game where misdirection, distraction, and visual illusiveness unfold on each play and the speed of the game dictates that variations in response execution on the order of 10s of milliseconds impact the success or failure of performance, *interference control* may be a key cognitive primitive among expert football players. Remarkably, while the best performing controls showed flanker effects on RT in the low 40 ms range, more than 20% of football athletes showed sub-40 ms flanker effects. Thus, a large percentage of football athletes demonstrate exceptional *interference control*. Moreover, of potential relevance to on-field performance is the degree of spread in *interference control* within the group of football athletes. Approximately, 53 ms separated the 10th and 90th percentiles of flanker effect performance within football athletes. From a mental chronometric standpoint, that difference is considerable (∼16% faster decision speed in the face of distraction) and likely to have significant impact in a reactively dynamic situation. As a comparison, consider the 40-yard dash times, a commonly-used metric to evaluate a football player’s foot speed, of two players. One runs an exceptionally fast 4.2 s 40-yard dash and the other runs the 40 in a modest 4.9 s, 16% slower. A speed difference between two runners of this magnitude will be readily discernible when they are running in the open field. Qualitatively, it is not unreasonable to speculate that individuals showing larger interference costs will be more prone to losing some response execution speed (i.e., to be tentative) in situations where conflicting response tendencies are activated concurrently by the visual misdirection movements of targets and surrounding opponent players, and this slowing will express itself in their play. Qualitative speculation aside, a central question for future research is establishing how much of a quantitative advantage or disadvantage *interference control* capabilities and speed afford on the football field.

Overall mean RTs were quite fast (in the 355 ms range) and indistinguishable between collegiate football players and age peers from the general student body. In fact, in the baseline condition in which the surrounding flankers pointed in the same direction as the target arrow, reactions were equally accurate and differed, on average, by just 4 ms between football athletes and age peers. These findings agree with those from a sub-set of studies in which comparable choice RTs have been reported between highly-skilled athletes who compete in visually-dynamic environments and controls (see references in Introduction). Thus, unlike professional badminton players whose overall RTs were faster (and tended to be faster to congruent arrays) than those of a highly-trained athletic control group (Wang et al., 2017), football athletes, despite possessing greater physical strength, speed, and athleticism as a group than typical age peers, may not possess an advantage in the overall speed or accuracy with which they execute the choice response decisions required of them in the Eriksen flanker task. Rather, football athletes as a group, unlike professional badminton players, demonstrate a specific advantage in shielding these response decisions from the interfering and conflicting response tendencies that are activated automatically by distracting stimulus events in the environment. In other words, higher-order cognitive control skills rather than basic decision and reaction speeds may distinguish football athletes as a group from age peers.

This skill difference may not be characteristic, however, of all football players, irrespective of position. A most intriguing finding was the demonstration of positional differences in *interference control* among football athletes. At the broadest level, defensive players show a significant advantage in *interference control* over offensive players, with both groups showing an advantage over their age peers. We reasoned in the Introduction that the ability to shield response execution from the interfering effects of distraction in the environment was a key cognitive skill for all football players, but that the demands on *interference control* might be higher for defensive than for offensive players. Defensive players, in their pursuit to defend against passes and tackle ball carriers, are challenged on each play by the efforts of the offense, both in coordinated group and in isolated individual player movements, to create unpredictability and misdirection. Thus, it is tempting to speculate that a successful defensive player must be able to minimize incorrect response tendencies and quickly resolve interference caused by processing the illusive and deceptive schemes of the offense in order to execute response decisions with speed and accuracy (i.e., to optimize his performance). Interestingly, all three defensive position groups (DL, LB, and DB) showed exceptional *interference control* skills. Worthy of note here is the finding by Williams et al. (2008) that elite defensive soccer players were superior to their offensive counterparts, and both were superior to novice defensive and offensive players, at anticipating opponent movements in a film-based anticipation test. In contrast to the defensive position group in our sample, only one offensive position group, WR/TE, showed enhanced interference control relative to their age peers. Here, it is tempting to speculate that, as the attackers, offensive players play with less uncertainty about their response execution decisions and encounter much less deceptive scheming from the defense. However, unlike their offensive teammates, WR/TE must make catches (1) in the face of a defender’s effort to block the pass or occlude the receiver’s vision of the pass, and (2) in congested areas of the field filled with the distractive movements of other players. Thus, the ability to focus attention and response execution on the target (i.e., the football) in the face of distraction may be a critical cognitive skill for receivers. It should be noted that while all of the remaining offensive positions showed numerically better *interference control* than age peers, the values were not statistically significant.

It may be surprising to the reader that quarterbacks were not clearly superior in *interference control* to either their teammates or age peers^3^. At first blush it would appear to be the case that a QB must be very adept at *interference control*. However, as we conceptualize the cognitive demands placed on a QB during the game when he sets up or drops back to pass, he must anticipate expanding his visual attentional field (i.e., broadening and distributing his attention) to attend to segments of the field where he expects his primary and then his secondary receiver to be, partition that attentional space into those sub-segments that separate the primary from the secondary receiver, initially direct his attention to the sub-segment where his primary receiver is expected to be, and determine whether or not he is open (i.e., there is a passing lane with sufficient distance separating the receiver from the defender(s) covering him). If the receiver is open, he passes the ball. If the receiver is not open, he shifts his attention to the sub-segment of his visual attentional field where the secondary receiver is expected to be. If that receiver is open, he passes the ball. If not, he shifts his attention to a tertiary receiver. This process typically unfolds in about 3000–4000 ms when the QB is given good protection and is allowed to stay in the pocket as he looks downfield. Integral to his decision-making process is broadening his visual attention to include defenders in the area near the receiver of interest and distributing his attention to track their movements in relation to that receiver. That is, he must attend primarily to the target (i.e., the receiver) while attending secondarily to the distractors (i.e., defenders) in his visual field, not inhibit the distractors. His decision to throw the football to the primary receiver or to look to the secondary receiver is determined, to a very important extent, by where the defenders are located relative to the receiver. Once he locates the defenders and judges there to be sufficient space between them and his receiver (i.e., determines there is a passing lane) he makes the decision to throw the ball. At this point, he quickly narrows his focus of attention on the receiver and delivers the pass. Moreover, while in the pocket he must be able to focus his visual attention downfield as he concurrently processes the actions of the on-coming rushers to step up into the pocket proficiently and to avoid a rush when necessary. Thus, the QB’s attention is routinely partitioned between the primary target and the secondary distractors in order to play his position most effectively.

The pattern of results that emerged in this study indicates that *interference control* is a cognitive skill that may be uniquely enhanced among a subset of collegiate American football players. While college athletes in this sport play at a very high level (i.e., one level removed from professional football), both a strength and a limitation of this study is that the results are novel and, consequently, the robustness of any conclusions drawn about how representative they are of DI football players and of potential NFL players must be enhanced and refined by replication. Concurrent with these replication efforts, it would be of value to initiate comparative assessments of professional football athletes. An important step in this direction has been taken by Solomon et al. (2013) in their comparison of elite NFL draft picks with then current NFL team roster players on a variety of neuropsychological, demographic, and medical/psychiatric variables. Among their findings, they reported that the draft picks had significantly faster visuomotor response times, as assessed by the ImPACT neuropsychological battery, than did the roster players. They did not attempt to relate their findings to on-field performance, however. This is a fundamental goal of research in this problem area and is, of course, the crown jewel for NFL coaches and front office personnel. Thus, determining direct linkages between neurocognitive skills, like *interference control*, and actual performance or performance statistics on the football field is essential to establishing the extent to which this type of research has both theoretical and applied value.

We know from about three decades of research that performance on the flanker task captures a critical component of human attention and executive control that provides meaningful translation to the detrimental impact of neurologic and psychiatric disease states on *interference control* (Ridderinkhof et al., 2005; Wylie et al., 2007, 2009a,b; Schmalbrock et al., 2016). From this research, we have also learned that deficits in *interference control* associated with different disease states are varied and complex. We know considerably less about factors that may be associated with superior *interference control* in the flanker task, but a nascent literature suggests that what constitutes high-level control will likewise be varied and complex. For example, more proficient control may be associated with (i) high levels of tested intelligence in adolescents (Liu et al., 2011, 2016); (ii) higher levels of aerobic fitness in preadolescents (Hillman et al., 2009; Moore et al., 2013; Berchicci et al., 2015; Westfall et al., 2017), adolescents (Hillman et al., 2006; Huang et al., 2015; but see Domazet et al., 2016), young, middle, and older adults (Hillman et al., 2006); (iii) bilingual fluency (Costa et al., 2008); and (iv) various types of cognitive expertise like that required to be a highly-skilled videogamer (Dye et al., 2009) or pilot (Roberts et al., 2010). What has emerged from this body of work is variability across studies in the dependent measures associated with superior *interference control*, which is suggestive of the complexity inherent in identifying patterns of superiority within and between various skill set domains.

An illustrative case in point of this complexity is the study by Roberts et al. (2010). These investigators assessed both the interference and facilitative effects induced by flankers in highly-trained fighter pilots on whom quite significant *interference control* demands are made. Using a version of the Eriksen flanker task in which subjects responded to vertical, rather than horizontal, arrow arrays, they found that pilots had comparable overall response speeds to controls but higher accuracy levels. In contrast to our findings and those of Wang et al. (2017), Roberts et al. (2010) found that the flanker interference effect was larger on RT in pilots than in controls and, in addition, pilots experienced a larger decrease in RT (i.e., facilitation) when the target was flanked by congruent arrows (as compared to a target flanked by neutral stimuli that did not signal a response, a condition not included in our study or in the Wang et al. study). In addition, they found that pilots had a larger pure cost of incongruence (slowing to incongruent relative to neutral arrays). They also analyzed paired trial-to-trial conflict relationships (difference between the RT on an *Ig* trial following an *Ig* trial and the RT on an *Ig* trial following a *Cg* trial) and found pilots to be more proficient than controls at modulating a response following an incongruent (i.e., conflict) trial. Thus, the response speeds of pilots were more influenced by the presence of flankers that signaled either the same or opposite response as the target than were those of controls, speeding up more in the first instance and slowing down more in the second instance. However, their performance accuracy was superior to that of controls in both instances and they were better able to modulate conflict effects than were controls.

Work like that of Roberts et al. (2010), Wang et al. (2017) and ours suggests that varied patterns of flanker effects are likely to be revealed across different sports, from perhaps no differences to dramatic differences between athletes and controls, and with those varied patterns the implications are likely to differ for the centrality of *interference control* in high-level performance within any given sport, from perhaps none to foundational. It also suggests the importance of probing the depths of the flanker effect in football players by doing, for example, trial-to-trial analyses like those done by Roberts et al. (2010) and extending that work to include distributional analyses on response speed and accuracy like those we and others have done in research with healthy adults and neurodegenerative disease (Wylie et al., 2007, 2009a,b; Forstmann et al., 2008; Davranche et al., 2009). Our current understanding of how *interference control* varies in different states and situations suggests that the magnitude of differences between the top and lower performing football players may be marked, perhaps quite specific, and very likely to influence, perhaps subtly, response execution efficiency on the field where subtle variations in decision speed, in the 10s of milliseconds, are likely to be critical to performance. Accordingly, it is important for future work to establish thresholds in *interference control* that *might* distinguish, for example, professional players from amateur players as well as elite professionals from all other professional players. It is also of fundamental importance to determine if *interference control* capabilities contribute to position profiles or handicap physical foot speed that then translate into practical applications in the sport of football.

Another fundamental issue for future research to address is determining the extent to which *interference control* capabilities among football players represent self-selected, hard-wired skills and/or are developed through experience in the football environment. That is, must an aspiring young football player possess an essential neurocognitive capacity like *interference control* in order to develop that particular skill through practice and training or is that basic capacity developed exclusively through practice and training? Existing research utilizing various imaging and electrophysiological techniques shows that variation in the proficiency of resolving response conflict among non-athlete healthy adults is linked to specific genetic influences, neuromodulators, and individual differences in patterns of neural activity in cognitive control circuitries (Kopp et al., 1996; Casey et al., 2000; Hazeltine et al., 2000; Bunge et al., 2002; Forstmann et al., 2008; Ochsner et al., 2009; Badgaiyan and Wack, 2011; Biehl et al., 2011; Newman et al., 2014; Chen et al., 2015). Outside of efforts to train these skills in children and, in particular, children with attention-deficit hyperactivity disorder, minimal work has focused on the trainability or modifiability of response conflict control skills in adults. We are aware of no work designed to sharpen these skills among highly-skilled athletes. Of note, Millner et al. (2012) showed that adults who trained on a related response conflict task (Simon task) experienced near-transfer effects on performance of the flanker conflict task. However, we are unaware of any studies that show training on response conflict tasks produces far-transfer effects that extend into a novel context like a sports setting. Interestingly, Colzato et al. (2013, 2014) showed that training on a first-person shooting video game produced transfer effects on other components of executive control (task switching, working memory) but not on response inhibition, and the beneficial effects were observed only in individuals with certain dopaminergic genetic predispositions. This pattern of results suggests that training cognitive control systems, including response conflict control, is likely to be complex, unique to various cognitive control systems, and vary on the basis of an individual’s genetic hardwiring and the specific nature of the training environment. Similarly, it is important to determine the extent to which peripheral sensory functions (e.g., vision) contribute to the development of high-level skill in a sport or only provide an important entry point for the conveyance of critical stimulus information to higher-order central neurocognitive systems, like those mediating *interference control*, that are essential to developing expert skill in any given sport (e.g., see Ward and Williams, 2003, who investigated these relationships in elite and sub-elite soccer players 9–17 years old).

The current study offers one of the first demonstrations that collegiate-level American football players possess an enhanced cognitive skill compared to their age peers, assessed in a basic laboratory task, and that this cognitive skill is not uniformly distributed across positions. A subset of, primarily defensive, football players may be more effective at shielding their response execution decisions from the interfering effects of distraction and incorrect response tendencies distraction elicits than their teammates or their age peers from the general student population. This difference in a crucial component of the brain’s cognitive control system may have important implications for making player selection decisions (e.g., recruiting), for finding the best player-position fit, and for developing individualized drill work to improve *interference control* capabilities during play.

## Author Contributions

SW, TB, NVW, and BA contributed to the study conception, design, and data acquisition. All authors (SW, TB, NVW, EM, KJ, JN, and BA) contributed to data analysis and interpretation and preparation of the manuscript. All authors approved of the final version to be published.

## Conflict of Interest Statement

The authors declare that the research was conducted in the absence of any commercial or financial relationships that could be construed as a potential conflict of interest.

## Footnotes

1 One of the reviewers raised a concern about differences in flanker effects related to handedness. We routinely collect self-reported hand dominance information from each participant in every study we run in our laboratories. The typical proportion of left-handers in any one given large sample (70–80 or more subjects) ranges from 8 to 10%. Unpublished comparative analyses we have done on left- and right-handed subjects in our Simon and Eriksen flanker task studies have revealed that among dextrals the response speeds of the dominant hand are typically faster than those of the non-dominant hand across factor levels, whereas among sinistrals there is a varied pattern of speed relationships. Some are faster with the dominant hand, some with the non-dominant hand, and some show no response-hand differences. Importantly, patterns of response accuracies associated with the different factor effects do not vary with response speed in either handedness group. The response-speed patterns we have found for both right- and left-handers are consistent with the patterns of strength of hand dominance known to exist among the two groups in the general population (see Coren, 1992). For reasons that are unknown, right-handers are commonly strongly right-hand dominant, whereas left-handers are commonly more mixed in their hand dominance. Of greatest importance is that our analyses have revealed no differences in factor effects or the directions of these effects between the two handedness groups. We searched the flanker-task literature and could not find a single paper in which handedness differences had been assessed.

2 https://cedrus.com/rb_series/

3 We did not address this issue in the original manuscript. It was raised in an insightful comment by one of the reviewers and catalyzed our thinking about the cognitive demands placed on a QB during a game in ways we had not previously considered. We are appreciative.

## References

[B1] Alves H. Voss M. W. Boot W. R. Deslandes A. Cossich V. Salles J. I. (2013). Perceptual-cognitive expertise in elite volleyball players. *Front. Psychol.* 4:36. 10.3389/fpsyg.2013.00036 23471100 PMC3590639

[B2] Badgaiyan R. D. Wack D. (2011). Evidence of dopaminergic processing of executive inhibition. *PLOS ONE* 6:e28075. 10.1371/journal.pone.0028075 22162756 PMC3230601

[B3] Berchicci M. Pontifex M. B. Drollette E. S. Pesce C. Hillman C. H. Di Russo F. (2015). From cognitive motor preparation to visual processing: the benefits of childhood fitness to brain health. *Neuroscience* 298 211–219. 10.1016/j.neuroscience.2015.04.028 25907444

[B4] Biehl S. C. Dresler T. Reif A. Scheuerpflug P. Deckert J. Herrmann M. J. (2011). Dopamine transporter (DAT1) and dopamine receptor D4 (DRD4) genotypes differentially impact on electrophysiological correlates of error processing. *PLOS ONE* 6:e28396. 10.1371/journal.pone.0028396 22162768 PMC3230585

[B5] Botvinick M. M. Braver T. S. Barch D. M. Carter C. S. Cohen J. D. (2001). Conflict monitoring and cognitive control. *Psychol. Rev.* 108 624–652. 10.1037/0033-295X.108.3.624 11488380

[B6] Bunge S. A. Hazeltine E. Scanlon M. D. Rosen A. C. Gabrieli J. D. (2002). Dissociable contribution of prefrontal and parietal cortices to response selection. *Neuroimage* 17 1562–1571. 10.1006/nimg.2002.1252 12414294

[B7] Burle B. Spieser L. Servant M. Hasbrouq T. (2014). Distributional reaction time properties in the Eriksen task: marked differences or hidden similarities with the simon task. *Psychn. Bull. Rev.* 21 1003–1010. 10.3758/s13423-013-0561-6 24302468 PMC4104006

[B8] Casey B. J. Thomas K. M. Welsh T. F. Badgaiyan R. D. Eccard C. H. Jennings J. R. (2000). Dissociation of response conflict, attentional selection, and expectancy with functional magnetic resonance imaging. *Proc. Natl. Acad. Sci. U.S.A.* 97 8728–8733. 10.1073/pnas.97.15.8728 10900023 PMC27016

[B9] Chang E. C.-H. Chu C.-H. Karageorghis C. I. Wang C.-C. Tsai J. H.-C. Wang Y.-S. (2017). Relationship between mode of sport training and general cognitive performance. *J. Sport Health Sci.* 6 89–95. 10.1016/j.jshs.2015.07.007 PMC6188876 30356524

[B10] Chen C. Yang J. Lai J. Li H. Yuan J. Abbasi N. (2015). Correlating gray matter volume with individual differences in the flanker interference effect. *PLOS ONE* 10:e0136877. 10.1371/journal.pone.0136877 26322974 PMC4554993

[B11] Colzato L. S. van den Wildenberg W. P. M. Hommel B. (2014). Cognitive control and the COMT Val158 met polymorphism: genetic modulation of videogame training and transfer to task-swithcing efficiency. *Psychol. Res.* 78 670–678. 10.1007/s00426-013-0514-8 24030137

[B12] Colzato L. S. van den Wildenberg W. P. M. Zmigrod S. Hommel B. (2013). Action video gaming and cognitive control: playing first person shooter games is associated with improvement in working memory but not action inhibition. *Psychol. Res.* 77 234–239. 10.1007/s00426-012-0415-2 22270615

[B13] Coren S. (1992). *The Left-Hander Syndrome: The Causes and Consequences of Left-Handedness.* New York, NY: Simon & Schuster.

[B14] Costa A. Hernaìndez M. Sebastiaìn-Galleìs N. (2008). Bilingualism aids conflict resolution: evidence from the ANT task. *Cognition* 106 59–86. 10.1016/j.cognition.2006.12.013 17275801

[B15] Davranche K. Hall B. McMorris T. (2009). Effect of acute exercise on cognitive control required during an eriksen flanker task. *J. Sport Exerc. Psychol.* 31 628–639. 10.1123/jsep.31.5.628 20016112

[B16] Domazet S. L. Tarp J. Huang T. Geji A. K. Andersen L. B. Froberg K. (2016). Associations of physical activity, sports participation and activie commuting on mathematic performance and inhibitory control in adolescents. *PLOS ONE* 11:e0146319. 10.1371/journal.pone.0146319 26727211 PMC4699746

[B17] Dye M. W. G. Green C. S. Bavelier D. (2009). The development of attention skills in action video game players. *Neuropsychologia* 47 1780–1789. 10.1016/j.neuropsychologia.2009.02.002 19428410 PMC2680769

[B18] Eriksen B. A. Eriksen C. W. (1974). Effects of noise letters upon the identification of a target letter in a nonsearch task. *Percept. Psychophys.* 16 143–149. 10.3758/BF03203267

[B19] Forstmann B. U. van den Wildenberg W. P. Ridderinkhof K. R. (2008). Neural mechanisms, temporal dynamics, and individual differences in interference control. *J. Cogn. Neurosci.* 20 1854–1865. 10.1162/jocn.2008.20122 18370596

[B20] Gratton G. Coles M. G. H. Sirevaag E. J. Eriksen C. W. Donchin E. (1988). Pre- and poststimulus activation of response channels: a psychophysiological analysis. *J. Exp. Psychol.* 14 331–344. 10.1037/0096-1523.14.3.331 2971764

[B21] Hazeltine E. Poldrack R. Gabrieli J. D. E. (2000). Neural activation during response competition. *J. Cogn. Neurosci.* 12 118–129. 10.1162/089892900563984 11506652

[B22] Hillman C. H. Buck S. M. Themanson J. R. Pontifex M. B. Castelli D. M. (2009). Aerobic fitness and cognitive development: event-related brain potential and task performance indices of executive control in preadolescent children. *Dev. Psycbol.* 45 114–129. 10.1037/a0014437 19209995

[B23] Hillman C. H. Motl R. W. Pontifex M. W. Posthuma D. Stubbe J. H. Boomsma D. I. (2006). Physical activity and cognitive function in a cross-section of younbger and older community-dwelling individuals. *Health Psychol.* 25 678–687. 10.1037/0278-6133.25.6.678 17100496

[B24] Huang T. Tarp J. Domazet S. L. Thorsen A. K. Froberg K. Andersen L. B. (2015). Associations of adiposity and aerobic fitness with executive function and math performance in danish adolescents. *J. Pediatr.* 167 810–815. 10.1016/j.jpeds.2015.07.009 26256018

[B25] Klein P. A. Petitjean C. Olivier E. Duque J. (2014). Top-down suppression of incompatible motor activations during response selection under conflict. *Neuroimage* 86 138–149. 10.1016/j.neuroimage.2013.08.005 23939021

[B26] Kokubu M. Ando S. Kida N. Oda S. (2006). Interference effects between saccadic and key-press reaction times of volleyball players and nonathletes. *Percept. Mot. Skills* 103 709–716. 10.2466/pms.103.3.709-716 17326494

[B27] Kopp B. Rist F. Mattler U. (1996). N200 in the flanker task as a neurobehavioral tool for investigating executive control. *Psychophysiology* 33 282–294. 10.1111/j.1469-8986.1996.tb00425.x 8936397

[B28] Liu T. Xiao T. Shi J. (2016). Fluid intelligence and neural mechanisms of conflict adaptation. *Intelligence* 57 48–57. 10.1016/j.intell.2016.04.003

[B29] Liu T. Xiao T. Shi J. Zhao D. Liu J. (2011). Conflict control of children with different intellectual levels: an ERP study. *Neurosci. Lett.* 490 101–106. 10.1016/j.neulet.2010.12.035 21182895

[B30] MathWorks (2014). *MATLAB and Statistics Toolbox Release.* Natick, MA: The MathWorks.

[B31] Mattler U. (2005). Flanker effects on motor output and the late-level response activation hypothesis. *Q. J. Exp. Psychol.* 58 577–601. 10.1080/02724980443000089 16104096

[B32] McDonald J. H. (2014). *Handbook of Biological Statistics*, 3rd Edn. Baltimore, MD: Sparky House Publishing, 140–144.

[B33] Memmert D. Simons D. J. Grimme T. (2009). The relationship between visual attention and expertise in sports. *Psychol. Sport Exerc.* 10 146–151. 10.1016/j.psychsport.2008.06.002

[B34] Millner A. J. Jaroszewski A. C. Chamarthi H. Pizzagalli D. A. (2012). Behavioral and electrophysiological correlates of training-induced cognitive control improvements. *Neuroimage* 63 742–753. 10.1016/j.neuroimage.2012.07.032 22836178 PMC3601637

[B35] Moore R. D. Wu C.-T. Pontifex M. B. O’Leary K. C. Scudder M. R. Raine L. B. (2013). Aerobic fitness and intra-individual variability of neurocognition in preadolescent children. *Brain Cogn.* 82 43–57. 10.1016/j.bandc.2013.02.006 23511845 PMC3632076

[B36] Mori S. Ohtani Y. Imanaka K. (2002). Reaction times and anticipatory skills of karate athletes. *Hum. Mov. Sci.* 21 213–230. 10.1016/S0167-9457(02)00103-3 12167300

[B37] Mori S. Shimada T. (2013). Expert anticipation from deceptive action. *Attent. Percept. Psychophys.* 75 751–770. 10.3758/s13414-013-0435-z 23436250

[B38] Mullane J. C. Corkum P. V. Klein R. M. McLaughlin E. (2009). Interference control in children with and without ADHD: a systematic review of Flanker and Simon task performance. *Child Neuropsychol.* 15 321–342. 10.1080/09297040802348028 18850349

[B39] Muraskin J. Sherwin J. Sajda P. (2015). Knowing when not to swing: EEG evidence that enhanced perception-action coupling underlies baseball batter expertise. *Neuroimage* 123 1–10. 10.1016/j.neuroimage.2015.08.028 26299795 PMC4626325

[B40] Newman D. P. Cummins T. D. Tong J. H. Johnson B. P. Pickering H. Fanning P. (2014). Dopamine transporter genotype is associated with a lateralized resistance to distraction during attention selection. *J. Neurosci.* 34 15743–15750. 10.1523/JNEUROSCI.2327-14.2014 25411502 PMC6608436

[B41] Ochsner K. N. Hughes B. Robertson E. R. Cooper J. C. Gabrieli J. D. (2009). Neural systems supporting the control of affective and cognitive conflicts. *J. Cogn. Neurosci.* 21 1842–1855. 10.1162/jocn.2009.21129 18823233 PMC6558970

[B42] Ridderinkhof K. R. (2002). “Activation and suppression in conflict tasks: Empirical clarification through distributional analyses,” in *Common Mechanisms in Perception and Action. Attention & Performance* Vol. 19 eds Prinz W. Hommel B. (Oxford: Oxford University Press), 494–519.

[B43] Ridderinkhof K. R. Scheres A. Oosterlaan J. Sergeant J. (2005). Delta plots in the study of individual differences: new tools reveal response inhibition deficits inAD/HD that are eliminated by methylphenidate treatment. *J. Abnorm. Psychol.* 114 197–215. 10.1037/0021-843X.114.2.197 15869351

[B44] Ridderinkhof K. R. van der Molen M. W. Bashore T. R. (1995). Limits on the application of additive factors logic: violations of stage robustness suggest a dual-process architecture to explain flanker effects on target processing. *Acta Psychol.* 90 29–48. 10.1016/0001-6918(95)00031-O

[B45] Roberts R. E. Anderson E. J. Husain M. (2010). Expert cognitive control and individual differences associated with frontal and parietal white matter microstructure. *J. Neurosci.* 30 17063–17067. 10.1523/JNEUROSCI.4879-10.2010 21159976 PMC3115511

[B46] Sanchez-Lopez J. Fernandez T. Silva-Pereyra J. Mesa J. A. M. Di Russo F. (2014). Differences in visuo-motor control in skilled vs. novice martial arts athletes during sustained and transient attention tasks: a motor-related cortical potential study. *PLOS ONE* 9:e91112. 10.1371/journal.pone.0091112 24621480 PMC3951282

[B47] Schmalbrock P. Prakash R. S. Schirda B. Janssen A. Yang G. K. Russell M. (2016). Basal ganglia iron in patients with multiple sclerosis measured with 7T quantitative susceptibility mapping correlates with inhibitory control. *Am. J. Neuroradiol.* 37 439–446. 10.3174/ajnr.A4599 26611996 PMC7960135

[B48] Solomon G. S. Haase R. F. Kuhn A. (2013). The relationship among neurocognitive performances and biopsychosocial characteristics of elite National Football League draft picks: an exploratory investigation. *Arch. Clin. Neuropsychol.* 28 9–20. 10.1093/arclin/acs108 23220623

[B49] van den Wildenberg W. P. Wylie S. A. Forstmann B. U. Burle B. Hasbroucq T. Ridderinkhof K. R. (2010). To head or to heed? Beyond the surface of selective action inhibition: a review. *Front. Hum. Neurosci.* 4:222. 10.3389/fnhum.2010.00222 21179583 PMC3004391

[B50] van Wouwe N. C. van den Wildenberg W. P. M. Claassen D. O. Kanoff K. Bashore T. R. Wylie S. A. (2014). Speed pressure in conflict situations impedes inhibitory action control in Parkinson’s disease. *Biol. Psychol.* 101 44–60. 10.1016/j.biopsycho.2014.07.002 25017503 PMC4504191

[B51] Verburgh L. Scherder E. J. A. van Lange P. A. M. Oosterlaan J. (2014). Executive functioning in highly talented soccer players. *PLOS ONE* 9:e91254. 10.1371/journal.pone.0091254 24632735 PMC3954684

[B52] Vestberg T. Gustafson R. Maurex L. Ingvar M. Petrovic P. (2012). Executive functions predict the success of top-soccer players. *PLOS ONE* 7:e34731. 10.1371/journal.pone.0034731 22496850 PMC3319604

[B53] Voss M. W. Kramer A. F. Basak C. Prakash R. S. Roberts B. (2010). Are expert athletes ‘expert’ in the cognitive laboratory? A meta-analytic review of cognition and sport expertise. *Appl. Cogn. Psychol.* 24 812–826. 10.1002/acp.1588

[B54] Wang C.-H. Chang C.-C. Liang Y.-M. Shih C.-M. Chiu W.-S. Tseng P. (2013). Open vs. closed sports and the modulation of inhibitory control. *PLOS ONE* 8:e55773. 10.1371/journal.pone.005773 23418458 PMC3572130

[B55] Wang C.-H. Yang C.-T. Moreau D. Muggleton N. G. (2017). Motor expertise modulates neural oscillations and temporal dynamics of cognitive control. *Neuroimage* 158 260–270. 10.1016/j.neuroimage.2017.07.009 28694229

[B56] Ward P. Williams A. M. (2003). Perceptual and cognitive skill development in soccer: the multidimensional nature of expert performance. *J. Sport Exerc. Psychol.* 25 93–111. 10.1123/jsep.25.1.93

[B57] Westfall D. R. Kao S.-C. Scudder M. R. Pontifex M. B. Hillman C. H. (2017). The association between aerobic fitness and congruency sequence effects in preadolescent children. *Brain Cogn.* 113 85–92. 10.1016/j.bandc.2016.12.005 28160688 PMC5346449

[B58] Williams A. M. Ward P. Smeeton N. J. Ward J. (2008). Task specificity, role, and anticipation skill in soccer. *Res. Q. Exerc. Sport* 79 429–433. 10.1080/02701367.2008.10599509 18816957

[B59] Wylie S. A. Ridderinkhof K. R. Eckerle M. K. Manning C. A. (2007). Inefficient response inhibition in individuals with mild cognitive impairment. *Neuropsychologia* 45 1408–1419. 10.1016/j.neuropsychologia.2006.11.003 17178419

[B60] Wylie S. A. van den Wildenberg W. P. M. Ridderinkhof K. R. Bashore T. R. Powell V. D. Manning C. A. (2009a). The effect of Parkinson’s disease on interference control during action selection. *Neuropsychologia* 47 145–157. 10.1016/j.neuropsychologia.2008.08.001 18761363 PMC4524676

[B61] Wylie S. A. van den Wildenberg W. P. M. Ridderinkhof K. R. Bashore T. R. Powell V. D. Manning C. A. (2009b). The effect of speed-accuracy strategy on response interference control in Parkinson’s disease. *Neuropsychologia* 47 1844–1853. 10.1016/j.neuropsychologia.2009.02.025 19428416 PMC4524649

[B62] Yamashiro K. Sato D. Onishi H. Sugawara K. Kakazawa S. Shimoto H. (2015). Skill-Specific changes in somatosensory nogopotentials in baseball players. *PLOS ONE* 10:e0142581. 10.1371/journal.pone.0142581 26600391 PMC4657892

